# Racial differences in diagnosis of perinatal mood and anxiety disorders

**DOI:** 10.1007/s00737-026-01713-2

**Published:** 2026-05-12

**Authors:** Stephanie V. Hall, Angela Montoya, Anca Tilea, Diana Louis, Kara Zivin, Vanessa K. Dalton

**Affiliations:** 1 Department of Psychiatry, University of Michigan Medical School, 2800 Plymouth Rd, Building 16, Ann Arbor, MI USA; 2 Program On Women’s Healthcare Effectiveness Research, Department of Obstetrics and Gynecology, University of Michigan Medical School, Ann Arbor, MI USA; 3 College of Human Medicine, Michigan State University, Lansing, MI USA; 4 Department of Obstetrics and Gynecology, University of Michigan Medical School, Ann Arbor, MI USA; 5 Department of Women’s and Gender Studies, College of Literature, Science, and the Arts, University of Michigan, Ann Arbor, MI USA; 6 Department of Health Policy and Management, School of Public Health, University of Michigan, Ann Arbor, MI USA; 7 VA Ann Arbor Healthcare System, Ann Arbor, MI USA; 8 Institute for Healthcare Policy and Innovation, University of Michigan, Ann Arbor, MI USA

**Keywords:** Diagnosis, Mental health, Perinatal mood and anxiety disorders, Racial differences

## Abstract

**Purpose:**

Perinatal mood and anxiety disorders (PMADs) represent a common comorbidity of pregnancy and postpartum with serious consequences. Despite high disease prevalence and burden, PMADs often remain undiagnosed. In recent years, universal screening for perinatal mental health disorders has become standard care and most patients are screened at least once during pregnancy or postpartum. Whether racial differences in PMAD diagnosis persist remains unknown. We aimed to measure and assess temporal trends in racial differences in PMAD diagnosis among symptomatic pregnant and postpartum Medicaid enrollees in Michigan.

**Methods:**

This serial cross-sectional observational study analyzed data from the Michigan Pregnancy Risk Assessment Monitoring System (MI-PRAMS) and Michigan Medicaid administrative claims for deliveries spanning 2016—2020. We linked MI-PRAMS responses to Medicaid claims from one year before through one year after delivery to determine whether enrollees who reported PMAD symptoms to MI-PRAMS received a PMAD diagnosis in Medicaid claims. We used weighted logistic regression to determine the association between race and PMAD diagnosis among Michigan Medicaid enrollees who reported PMAD symptoms.

**Results:**

Out of 60,583 Medicaid enrollees with a live birth in Michigan from 2016—2020, 33% were Black and 61% were White. About 70% were between 19—29 years old and 18% were between 30—34 years old. During the perinatal period, 39% had 2 or more co-morbid conditions, and 60% had 2 or more major life stressors. In unadjusted weighted logistic regression, White enrollees had 2.146 times greater odds of PMAD diagnosis than Black enrollees (95% CI: 1.570—2.933). This association remained significant after adjusting for age, ethnicity, co-morbidities, major life stressors, and delivery year. Adjusted weighted logistic regression found that White enrollees had 2.326 times greater odds of PMAD diagnosis than Black enrollees (95% CI: 1.669—3.236).

**Conclusion:**

White enrollees who reported PMAD symptoms were more than twice as likely to receive a PMAD diagnosis as Black enrollees reporting PMAD symptoms. The magnitude of racial difference in PMAD diagnosis did not improve over the course of the study period. These persistent racial differences are consistent with structural barriers, provider biases, and cultural stigmas that differentially and disproportionately impact non-White patients. Tailored interventions to improve mental health care among Black Medicaid enrollees may be needed to improve PMAD diagnosis, a critical first step in mental health management.

**Clinical trial number:**

Not applicable.

## Highlights

We linked self-reported symptoms of perinatal mood and anxiety disorder to clinical diagnoses of perinatal mood and anxiety disorder to determine differences in diagnosis rate.

Among Medicaid enrollees reporting symptoms of perinatal mood and anxiety disorder, White enrollees were more than twice as likely to receive a diagnosis of perinatal mood and anxiety disorder compared to Black enrollees.

The magnitude of racial difference in odds of perinatal mood and anxiety disorder diagnosis did not significantly change over the study period.

## Introduction

Perinatal mood and anxiety disorders (PMADs) represent a common comorbidity of pregnancy with serious consequences [1, 2]. PMADs affect one in five deliveries and increase the risk of adverse outcomes including low birthweight, preterm birth, and severe maternal morbidity (SMM) [3–8]. Suicide has become a leading cause of preventable maternal mortality [9]. PMADs increase health care utilization and costs [5, 10] while incurring societal costs of more than $14 billion annually in the United States [11].

Despite high disease prevalence and burden, PMADs often remain unrecognized and undiagnosed. Cox et al. estimate that only 49.9% of antenatal PMAD cases receive a diagnosis while only 30.8% of postpartum PMAD cases receive a diagnosis [12]. Underdiagnosis of PMAD may inhibit adequate treatment, lead to unmanaged PMAD symptoms, and exacerbate the adverse maternal and infant outcomes associated with PMAD [12]. Diagnosis is therefore a critical step toward clinical management of PMAD.

Between 2014 and 2018, several clinical initiatives and policy changes aimed to improve access and standardize mental health screenings in obstetric care. In 2015, the American College of Obstetrics and Gynecology (ACOG) issued recommendations for universal mental health screening during the perinatal period [13]. The U.S. Preventive Services Task Force (USPSTF) issued similar recommendations in 2016 [14]. Furthermore, beginning in 2014, the Patient Protection and Affordable Care Act (ACA) reduced administrative and financial barriers to both obstetric and mental health care. [15]

There is evidence that these efforts increased perinatal mental health screening, as PMAD diagnoses climbed steadily from 2008 through 2020 [8]. By 2016, nearly 90% of obstetricians reported routinely screening all pregnant and postpartum patients for depression [16]. In 2018, 80% of women reported having a health care provider discuss depression during a prenatal visit while almost 90% reported having a health care provider discuss depression during a postpartum visit [17]; however, these rates varied tremendously by state [17].

Assessing the prevalence of PMAD underdiagnosis remains methodologically difficult and risk factors for underdiagnosis remain largely unexplored [18–20]. Literature on the existence of racial differences in PMAD underdiagnosis is mixed [21, 22]. For example, our prior work identified large differences in postpartum clinical diagnoses between Black and White Michigan Medicaid enrollees who delivered between 2012 to 2015 [21], prior to universal mental health screening recommendations [13]. In an analysis of 2020 deliveries spanning multiple regions across the United States, postpartum diagnosis rates did not statistically differ by race [22]. Updated estimates of underdiagnosis are needed to determine if clinical and policy initiatives have helped to close racial differences in perinatal mental health diagnosis.

Despite the rapid uptake of clinical and policy initiatives to improve PMAD screening, the impact of these efforts on racial differences in PMAD diagnosis remains unknown [23, 24]. This study aimed to determine if racial differences in PMAD diagnosis among symptomatic Medicaid enrollees who delivered between 2016 and 2020 improved over time. We hypothesized that the changing clinical, policy, and cultural landscape of perinatal mental health leading to increased screening may have disproportionately improved detection among Black Medicaid enrollees and reduced differences in PMAD diagnosis.

## Materials and methods

This serial cross-sectional observational study analyzed data from the Michigan Pregnancy Risk Assessment Monitoring System (MI-PRAMS) linked to Michigan Medicaid administrative claims. Respondents delivered between 2016 and 2020 and the corresponding perinatal Medicaid claims for these deliveries spanned 2015 through 2021. The Michigan Department of Health and Human Services (MDHHS) administers the MI-PRAMS survey in coordination with the Centers for Disease Control and Prevention (CDC) on an annual basis [25]. MDHHS conducts stratified sampling of birth certificates to identify a representative sample of recent deliveries and surveys individuals by mail or phone 2—6 months after delivery [26]. Michigan Medicaid administrative claims include diagnoses, procedures, and related health services billed to Medicaid. In coordination with partners at MDHHS, we linked MI-PRAMS and Michigan Medicaid claims at the individual level to produce a representative sample of delivering Michigan Medicaid enrollees with both MI-PRAMS responses and continuous Medicaid enrollment in the year before and after delivery.

We limited the linked data to Medicaid enrollees with PMAD symptoms on the MI-PRAMS survey. We defined PMAD symptoms as either (1) affirming depression during pregnancy or (2) screening positive for postpartum depressive symptoms on the two-item patient health questionnaire survey (PHQ-2) [27]. The PHQ-2 asks how often respondents “feel down, depressed, or hopeless” or have “little interest or pleasure in doing things” on a five-point scale of “never, rarely, sometimes, often, or always.” Respondents who respond “often” or “always” to either item screen positive for postpartum depressive symptoms.

### Outcome variable

The outcome of interest was a clinical diagnosis of mood or anxiety disorder during the perinatal period defined as one year before delivery through one year after delivery. We defined mood or anxiety disorder diagnoses based on International Classification of Disease Tenth Revision (ICD-10) codes drawn from the Health Care Utilization Project (HCUP) and shown in Appendix A [28]. ICD-10 was the diagnostic classification used in clinical care for all years of data. We required diagnoses to appear in at least one inpatient claim or two outpatient claims during the perinatal period to minimize misclassification.

### Independent variables

The primary independent variable of interest was race. This dataset includes racial categories of White, Black, Asian, and Other/Unknown. Our primary analysis includes only Black and White enrollees due to small cell counts in other racial categories. We also evaluated whether ethnicity, age, comorbidities, major life stressors in the year prior to pregnancy, preterm birth, severe maternal morbidity (SMM), and cesarean delivery were associated with diagnosis.

We dichotomized ethnicity as Hispanic or non-Hispanic. We categorized age as under 19, 19—24, 25—29, 30—34, 35—39, or ≥ 40 years of age at the time of delivery for descriptive statistics and included age as a continuous variable in statistical models. We used the Obstetric Comorbidity Index (OBCMI) to measure comorbidities [29]. The OBCMI is a composite score of obstetric health factors such as body mass index, diabetes status, or other diagnoses. Higher OBCMI scores indicate greater risk of severe maternal morbidity (SMM), defined as a list of 21 critical adverse events that occur during or after delivery [30]. OBCMI scores are typically left-skewed with a low median score of approximately 2 [29]. We dichotomized the OBCMI score as 0—1 and 2 + to reflect this distribution, as done in prior literature. Major life stressors in the year before pregnancy include social, emotional, and financial stressors such as divorce, death of a loved one, or job loss. We dichotomized the number of life stressors as 0—2 or 3 +. We used ICD and procedural codes to assess preterm birth, defined as delivery before 37 weeks gestation, cesarean delivery, SMM within 42 or 365 days of delivery.

### Statistical approach

We used weighted frequencies to describe the delivering cohort and survey-weighted logistic regression to model odds of PMAD diagnosis. For adjusted models we controlled for age, race, ethnicity, comorbidities, life stressors, and year of delivery.

We conducted a sensitivity analysis including an interaction term between race and year of delivery to determine if the association between race and PMAD diagnosis changed over the course of the study period.

Our main analysis examined the entire perinatal period of the year before delivery through the year after delivery; however, we conducted sensitivity analyses to separately evaluate the year before and the year after delivery to determine if there were differences in antenatal and postpartum trends.

All statistical analyses were conducted using SAS, version 9.4 (Cary, NC). [31]

The Institutional Review Boards at both the University of Michigan (HUM00148854) and the Michigan Department of Health and Human services (#201,811—10-EA) approved this research study.

## Results

**Fig. 1:**
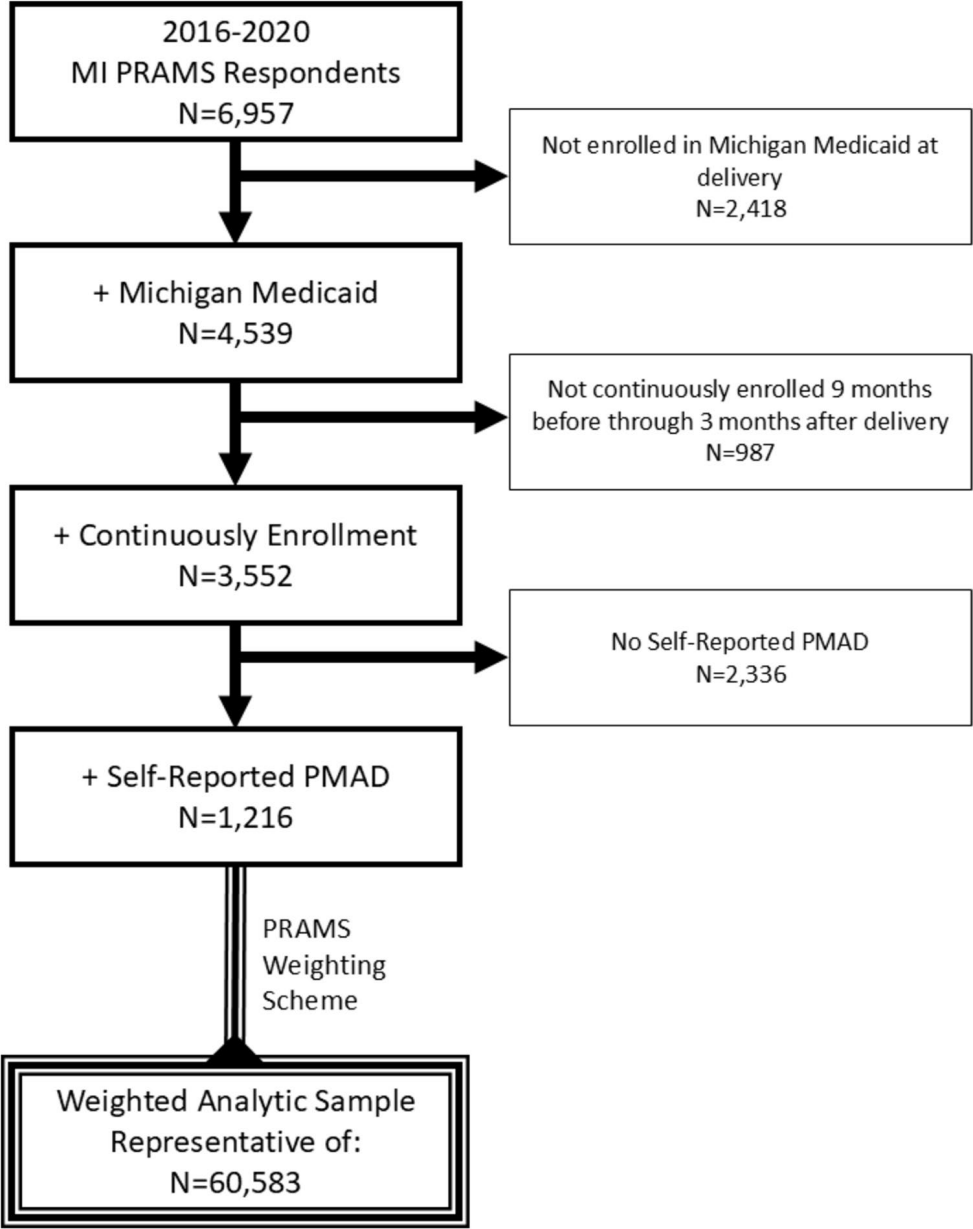
Study cohort diagram

A total of 1,216 delivering Michigan Medicaid enrollees responded to the MI-PRAMS survey with PMAD symptoms between 2016 and 2020. Their responses represent 60,583 delivering Michigan Medicaid enrollees with PMAD symptoms when weighted according to the MI-PRAMS weighting scheme. [32] A study cohort diagram appears in Fig. 1.

The weighted sample included 20,535 Black enrollees and 37,167 White enrollees. Sociodemographic characteristics were similar across Black and White enrollees. There were no statistically significant differences in age, percent with OBCMI ≥ 2, percent with ≥ 2 major life stressors, preterm birth, cesarean birth, or SMM. Overall, 38.0% of enrollees with PMAD symptoms had a PMAD diagnosis. The PMAD diagnosis rate was significantly different across racial groups; 44.5% of White enrollees had a PMAD diagnosis compared to only 27.2% of Black enrollees (p < 0.001). Table 1 presents sociodemographic characteristics overall and for Black and White racial subgroups.

**Table 1:** Sociodemographic characteristics among delivering Medicaid enrollees with PMAD symptoms, overall and stratified by Black and White racial subgroups

	Overall (n = 60,583)		Black (n = 20,535)		White (n = 37,167)		p-value
Demographics	n	%	n	%	n	%	
PMAD Diagnosis	23,017	38.0%	5582	27.2%	16,528	44.5%	0.0001
Age group							
< 19	2191	3.6%	1033	5.0%	1158	3.1%	0.3763
19—24	21,256	35.1%	6819	33.2%	13,624	36.7%	
25—29	20,772	34.3%	6706	32.7%	12,762	34.3%	
30—34	10,912	18.0%	3999	19.5%	6561	17.7%	
35—39	4511	7.4%	1571	7.6%	2767	7.4%	
40 +	941	1.6%	407	2.0%	295	0.8%	
Obstetric Comorbidity Index (≥ 2)	23,634	39.0%	8774	42.7%	13,637	36.7%	0.2929
Preterm Birth	9507	15.7%	3723	18.1%	5247	14.1%	0.2792
Cesarean Birth	20,003	33.0%	6985	34.0%	11,882	32.0%	0.6383
SMM within 365 Days of Delivery	1174	1.9%	540	2.6%	588	1.6%	0.4127
SMM Within 42 Days of Delivery	1401	2.3%	579	2.8%	800	2.2%	0.4117
Major Life Stressors (≥ 3)	35,798	59.1%	11,700	57.0%	22,809	61.4%	0.1511

**Table 2:** Adjusted and unadjusted odds of PMAD diagnosis among delivering Medicaid enrollees with PMAD symptoms

	Unadjusted Odds Ratio	95% Confidence Interval	Adjusted Odds Ratio	95% Confidence Interval
White (ref: Black)	**2.146**	**1.570—2.933**	**2.326**	**1.669—3.236**
Age at delivery (years)			1.017	0.984—1.051
Hispanic (ref: Non-Hispanic)			0.968	0.396—2.364
OBCMI ≥ 2 (ref: < 2)			**1.816**	**1.248—2.642**
> 3 Stressors (ref: < 3)			**1.645**	**1.124—2.408**
Delivery year			0.953	0.838—1.084

**Fig. 2:**
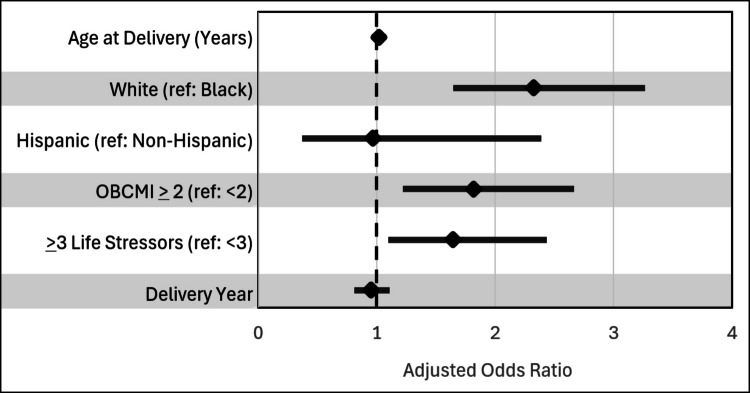
Adjusted odds ratio for PMAD diagnosis among delivering Medicaid enrollees with PMAD symptoms

Unadjusted weighted logistic regression found that White enrollees with PMAD symptoms had 2.146 greater odds of PMAD diagnosis than Black enrollees with PMAD symptoms (95% CI: 1.570—2.933). This association remained significant after adjusting for delivery year, ethnicity, OBCMI, and age. Adjusted weighted logistic regression found that White enrollees with PMAD symptoms had 2.326 greater odds of PMAD diagnosis than Black enrollees with PMAD symptoms (95% CI: 1.669—3.236). We also found that enrollees with OBCMI scores ≥ 2 had 1.816 times greater odds of PMAD diagnosis than enrollees with OBCMI scores of 0—1 (95% CI: 1.248—2.642). Enrollees with 3 or more life stressors in the year prior to pregnancy had 1.645 times greater odds of PMAD diagnosis than those with 2 or fewer life stressors (95% CI: 1.124—2.408). Logistic regression models appear in Table 2 and a corresponding forest plot of adjusted odds ratios appears in Fig. 2.

**Fig. 3:**
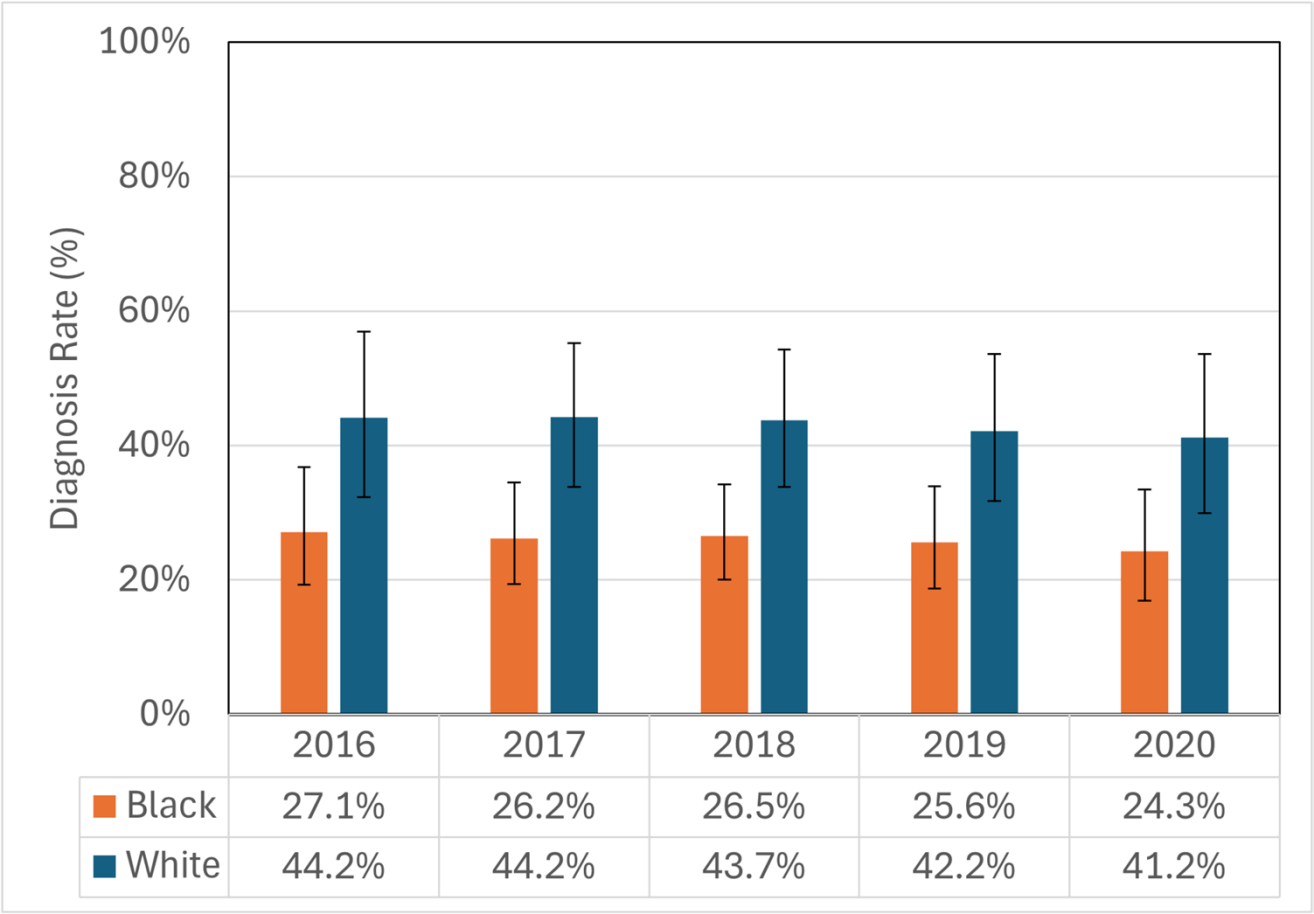
Adjusted PMAD diagnosis rate among delivering Medicaid enrollees with PMAD symptoms

Figure 3 shows the yearly predicted probability of PMAD diagnosis by race. The adjusted PMAD diagnosis rate, measured by the predicted probability of diagnosis and drawn from the adjusted regression model, was significantly higher among White enrollees than Black enrollees. Among White enrollees with PMAD symptoms, the percent with a PMAD diagnosis declined from 44.2% in 2016 to 41.2% in 2020. Among Black enrollees with PMAD symptoms, the diagnosis rate declined from 27.1% in 2016 to 24.3% in 2020. White enrollees had a higher rate of PMAD diagnosis than Black enrollees in all years.

Sensitivity analyses that included an interaction term between race and delivery year (data not shown) found no significant interaction between race and delivery year (p > 0.05), indicating that the racial difference in odds of diagnosis did not significantly change over the study period. Sensitivity analyses that separately examined the year before delivery and the year after delivery (data not shown) also found that White enrollees with PMAD symptoms were more likely to receive a PMAD diagnosis than Black enrollees with PMAD symptoms. Sensitivity analyses that included an interaction term between race and ethnicity (data not shown) produced similar results.

## Discussion and conclusions

Among delivering Michigan Medicaid enrollees with self-reported PMAD symptoms during pregnancy and postpartum, White enrollees were more than twice as likely to receive a PMAD diagnosis as Black enrollees. Since all included enrollees reported PMAD symptoms, the racial difference in diagnosis rates do not reflect a difference in disease prevalence; rather, the racial difference in diagnosis rate suggests that other factors drive higher rates of missed diagnosis in Black enrollees. Contributing factors such as provider bias, stigma, or differential access to care may create racial difference in the detection or documentation of mental health disorders among obstetric patients. Our findings suggest that recent clinical and policy efforts to increase PMAD screening were insufficient to address racial differences in identification and treatment of PMAD.

These findings are consistent with our prior work, which found delivering Michigan Medicaid enrollees with self-reported PMAD symptoms experienced a similar racial difference in postpartum PMAD diagnosis from 2012—2015 [21]. In 2015 and 2016, both ACOG and the USPSTF recommended universal perinatal depression screening [13, 14]. Despite widespread uptake of universal screening [16, 17], these data for Medicaid enrollees who delivered from 2016—2021 found a similar racial difference in PMAD diagnosis across all years of analysis and throughout the perinatal period. Notably, the non-significant interaction term between race and year of delivery indicates that racial difference in diagnosis remained unchanged over time.

Few other studies have assessed PMAD underdiagnosis due to the challenges of detecting undetected PMAD. Haight et al. conducted a follow-up survey of PRAMS enrollees in seven jurisdictions to determine whether enrollees who screened positive on the initial PHQ-2 screening questionnaire received a PMAD diagnosis by 12-months postpartum [22]. Although Haight et al. identified racial differences in PMAD treatment, they did not detect racial differences in self-reported PMAD diagnosis. Haight et al. asked respondents if they were diagnosed with PMAD by a health care provider; however, it’s possible respondents also used this question to self-report PMAD, whereas our study used Medicaid claims to identify PMAD diagnoses made by health care providers. The lack of racial difference in self-reported PMAD diagnosis may reflect this difference in measurement. Self-reported PMAD represents an accurate measure of depression due to patients’ ability to self-identify symptoms [33], whereas clinical diagnoses are filtered through multiple steps, including access to care, screening, detection, and documentation.

The observed racial differences in PMAD diagnosis reflect upstream differences in maternal health care. For example, in a large healthcare system in the midwestern United States, Black obstetric patients were about 20% less likely to be screened for mental health disorders during the perinatal period than White obstetric patients despite universal screening guidelines [34] and Black PRAMS respondents were less likely to report receiving mental health screening during the recommended postpartum check-up than White PRAMS respondents [35]. Black women are also less likely to receive routine prenatal and postpartum care [36]. These differences create fewer opportunities for health care providers to diagnose mental health symptoms. In addition to quantitative differences in care, the quality of care may also differ [37, 38] as Black women are less likely to receive culturally sensitive clinical care [39] and less likely to trust their providers. [40]

Our data cannot identify the causal factors that lead to racial differences in obstetric mental health care. However, literature indicates that these findings may be driven by larger misalignment of mainstream medicine with common cultural conceptions related to Black mental health. The “strong black woman” remains a pervasive schema, which implies Black women can “tolerate the intolerable” [41] due to strength and invulnerability [42]. This schema remains incongruent with the aims, practices, and modes of mainstream medicine, and may impede mental health care for Black women through many mechanisms. Providers may assume biological differences engender Black patients with higher pain thresholds than White patients [43] while Black patients may downplay or avoid disclosing symptoms in order to portray this assumed strength or eschew negative stereotypes [44]. The incongruence of mainstream medicine and cultural conceptions of Black mental health may lead Black mothers to reject mental health treatment at higher rates than White mothers, especially regimens that emphasize medication [45]. While mainstream medicine is oriented toward the individual, Black women are more inclined toward communal ways of dealing with mental health, especially those that emphasize spiritual traditions. [44, 46]

Lower rates of PMAD diagnosis among symptomatic Black Medicaid enrollees have major implications for adequate mental health care and may exacerbate downstream outcomes. Without diagnosis, Black patients may be less likely to receive mental health care until symptoms become life-threatening. From 2008—2018, antenatal depression diagnoses increased 66% among Black commercially insured beneficiaries while antenatal suicidality increased more than 700% [47]. Similarly, Black commercially insured beneficiaries experienced the greatest increase in perinatal suicidality from 2006—2017 [48]. Diagnosis is a critical precursor to treatment, and without treatment, symptoms go unmanaged and increase the odds of reaching a crisis point.

Our findings provide additional context to research indicating widespread racial differences in PMAD treatment among those with a PMAD diagnosis [22, 49–51]. Our results suggest that racial differences in care begin, even prior to treatment considerations, at the diagnostic stage. This would indicate that racial differences in treatment initiation among those with PMAD diagnoses may be even larger than previously reported because Black patients are underrepresented among patients with PMAD diagnoses.

Improved mental health screening, detection, diagnosis, and treatment among Black obstetric patients represent critical steps toward improving outcomes. Universal guidelines have increased diagnosis rates across the population, but precision approaches may be needed to improve diagnosis rates among underserved populations. For example, although ACOG recommends universal screening at least one time point during the perinatal period, additional targeted screening of populations at risk for underdiagnosis and tailored protocols that specifically address the existing barriers to diagnosis could promote obstetric mental health care among Black patients and assuage the racial differences observed in this study. Culturally sensitive training, a diverse workforce, and non-stigmatizing approaches to mental health may further support discussion regarding mental health in clinical settings.

### Strengths and limitations

This study is strengthened by the individual-level linkage of multiple data sources to triangulate and compare symptoms and clinical diagnoses. MI-PRAMS is a rigorous and representative survey while Medicaid claims include all health care interactions billed to Medicaid. Despite these strengths, some weaknesses may limit this study. MI-PRAMS is a cross-sectional survey and may be subject to recall, non-response, and social desirability bias. Similarly, while Medicaid data provide a comprehensive record of billed diagnoses and procedures, enrollees who seek mental health care (e.g., therapy) outside Medicaid coverage may be less likely to have mental health disorders documented in clinical records that are billed to Medicaid. We do not know the true prevalence of clinical PMADs and rely on self-reported status and PHQ-2 score as a proxy, which could create misclassification bias. Data only represent deliveries through 2020 and postpartum health care receipt through 2021. Some respondents delivered or received postpartum health care after the onset of the COVID-19 pandemic. Although the COVID-19 pandemic may have impacted mental health or health care for some enrollees in these data, the societal effects of the COVID-19 pandemic do not appear to significantly impact the results, as the temporal trend in diagnosis rates does not deviate for deliveries from 2020 compared to 2016—2019. Sensitivity analyses excluding data from 2020 and beyond produced similar results; however, these findings may not generalize to post-pandemic years.

## Conclusions

Accurate and timely diagnosis of PMAD represents a critical first step in perinatal mental health management. The racial difference in PMAD diagnosis indicates that Black women experiencing PMADs may not have access to adequate treatment or be represented in health data. These findings reinforce the pervasive trend that perinatal health care often fails Black women. Tailored efforts to identify and address the underlying drivers of these differences are needed to improve quality of care and health outcomes for Black women.

## Data Availability

The data analyzed in this study are not publicly available because these data were obtained from the Michigan Department of Health and Human Services (MDHHS) under a Data Use Agreement (DUA) that restricts public sharing of data.

## Appendix A

**Table 3:** International Classification of Disease Tenth Revision (ICD-10) codes used to identify maternal mental health disorders

Group	ICD Diagnosis Code
Anxiety	300.xx, 308.xx, 313.xx, 293.xx, F06.xx, F40.xx, F41.xx, F42.xx, F43.xx, F48.xx, R45.xx
Depression	311.xx, 296.xx, 300.xx, F32.xx, F33.xx

## Declarations

### Data

We thank the Pregnancy Risk Assessment Monitoring System (PRAMS) Working Group, which includes the PRAMS Team, Division of Reproductive Health, Centers for Disease Control and Prevention (CDC) and the Michigan PRAMS site for their role in conducting PRAMS surveillance and allowing the use of their data.

## References

[CR1] 111th Congress. The Patient Protection and Affordable Care Act (PPACA). Public Law 111–148, 124 Stat 119 (March 23, 2010)2010.

[CR2] SAS Institute. *SAS 9.4 SQL *Procedure* User's Guide*. SAS Institute; 2015.

[CR3] Admon LK, Dalton VK, Kolenic GE et al (2020) Trends in Suicidality 1 Year Before and After Birth Among Commercially Insured Childbearing Individuals in the United States, 2006–2017. JAMA Psychiat 78(2):171–176. 10.1001/jamapsychiatry.2020.3550 10.1001/jamapsychiatry.2020.3550 PMC7675215 33206140

[CR4] Agency for Healthcare Research and Quality. HCUP CCS-Services and Procedures. Healthcare Cost and Utilization Project (HCUP). 2018.

[CR5] American College of Obstetricians and Gynecologists. ACOG Committee Opinion No. 630: Screening for Perinatal Depression. 2015. 10.1097/01.AOG.0000465192.34779.dc 25932866

[CR6] Bauman BL, Ko JY, Cox S et al (2020) Vital signs: postpartum depressive symptoms and provider discussions about perinatal depression - United States, 2018. MMWR Morb Mortal Wkly Rep 69(19):575–581. 10.15585/mmwr.mm6919a2 32407302 10.15585/mmwr.mm6919a2 PMC7238954

[CR7] Beauboeuf-Lafontant T (2005) Keeping up appearances, getting fed up: the embodiment of strength among African American women. Meridians Middletown Conn 5(2):104–123. 10.1353/mer.2005.0003

[CR8] Burton A, Patel S, Kaminsky L et al (2011) Depression in pregnancy: time of screening and access to psychiatric care. J Matern Fetal Neonatal Med 24(11):1321–1324. 10.3109/14767058.2010.547234 21261444 10.3109/14767058.2010.547234

[CR9] Conteh N, Gagliardi J, McGahee S, Molina R, Clark CT, Clare CA (2022) Medical mistrust in perinatal mental health. Harv Rev Psychiatry 30(4):238–247 35849741 10.1097/HRP.0000000000000345

[CR10] Cousin L, Johnson-Mallard V, Booker SQ (2022) “Be strong my sista’”: sentiments of strength from Black women with chronic pain living in the Deep South. Adv Nurs Sci 45(2):127–142. 10.1097/ANS.0000000000000416 10.1097/ANS.0000000000000416 PMC9064901 35234672

[CR11] Cox EQ, Sowa NA, Meltzer-Brody SE, Gaynes BN (2016) The perinatal depression treatment cascade: baby steps toward improving outcomes. J Clin Psychiatry 77(9):1189–1200. 10.4088/JCP.15r10174 27780317 10.4088/JCP.15r10174

[CR12] Easter SR, Bateman BT, Sweeney VH et al (2019) A comorbidity-based screening tool to predict severe maternal morbidity at the time of delivery. Am J Obstet Gynecol 221(3):271 e1-271 e10. 10.1016/j.ajog.2019.06.025 31229427 10.1016/j.ajog.2019.06.025

[CR13] Gavin NI, Gaynes BN, Lohr KN, Meltzer-Brody S, Gartlehner G, Swinson T (2005) Perinatal depression: A systematic review of prevalence and incidence. Obstet Gynecol 106(5 Part 1):1071–1083. 10.1097/01.AOG.0000183597.31630.db 16260528 10.1097/01.AOG.0000183597.31630.db

[CR14] Goodman JH, Tyer-Viola L (2010) Detection, treatment, and referral of perinatal depression and anxiety by obstetrical providers. J Womens Health Larchmt 19(3):477–490. 10.1089/jwh.2008.1352 20156110 10.1089/jwh.2008.1352

[CR15] Haight SC, Daw JR, Martin CL et al (2024) Racial and ethnic inequities in postpartum depressive symptoms, diagnosis, and care in 7 US jurisdictions. Health Aff 43(4):486–495. 10.1377/hlthaff.2023.01434 10.1377/hlthaff.2023.01434 PMC13034582 38560804

[CR16] Hall SV, Pangori A, Tilea A, Schroeder A, Admon LK, Zivin K (2024) Antidepressant prescriptions increased for privately insured people with perinatal mood and anxiety disorder, 2008-20. Health Aff Millwood 43(4):514–522. 10.1377/hlthaff.2023.01448 38560803 10.1377/hlthaff.2023.01448 PMC11164068

[CR17] Hall SV, Zivin K, Piatt GA et al (2024) Racial disparities in diagnosis of postpartum mood and anxiety disorders among symptomatic Medicaid enrollees, 2012–2015. Psychiatric Services (Washington, DC) 75(2):115–123. 10.1176/appi.ps.20230094 10.1176/appi.ps.20230094 37752825

[CR18] Hall SV, Zivin K, Piatt GA et al (2023) Factors associated with mental health treatment among Michigan Medicaid enrollees with perinatal mood and anxiety disorders, 2012–2015. Gen Hosp Psychiatry 83:164–171. 10.1016/j.genhosppsych.2023.05.009 37210824 10.1016/j.genhosppsych.2023.05.009

[CR19] Hoffman KM, Trawalter S, Axt JR, Oliver MN (2016) Racial bias in pain assessment and treatment recommendations, and false beliefs about biological differences between Blacks and Whites. Proc Natl Acad Sci U S A 113(16):4296–4301. 10.1073/pnas.1516047113 27044069 10.1073/pnas.1516047113 PMC4843483

[CR20] Ibrahim BB, Vedam S, Illuzzi J, Cheyney M, Kennedy HP (2022) Inequities in quality perinatal care in the United States during pregnancy and birth after cesarean. PLoS One 17(9):e0274790 36137150 10.1371/journal.pone.0274790 PMC9499210

[CR21] Interrante JD, Admon LK, Carroll C, Henning-Smith C, Chastain P, Kozhimannil KB (2022) Association of health insurance, geography, and race and ethnicity with disparities in receipt of recommended postpartum care in the US. JAMA Health Forum 3(10):e223292–e223292. 10.1001/jamahealthforum.2022.3292 36239954 10.1001/jamahealthforum.2022.3292 PMC9568809

[CR22] Karasek D, Collin DF, Hamad R, Jackson K, Gemmill A (2025) Perinatal health and healthcare utilisation during the COVID‐19 pandemic: A nationwide interrupted time series analysis. Paediatr Perinat Epidemiol 39(4):373–384. 10.1111/ppe.70000 40075540 10.1111/ppe.70000 PMC12121338

[CR23] Kozhimannil KB, Trinacty CM, Busch AB, Huskamp HA, Adams AS (2011) Racial and ethnic disparities in postpartum depression care among low-income women. Psychiatric Services (Washington, DC) 62(6):619–625. 10.1176/appi.ps.62.6.619 10.1176/appi.ps.62.6.619 PMC3733216 21632730

[CR24] Leboffe EN, Pietragallo HC, Liu G, Ba D, Leslie D, Chuang CH (2023) The impact of the 2015 ACOG screening guidelines on the diagnosis of postpartum depression among privately insured women. J Affect Disord 328:103–107. 10.1016/j.jad.2023.02.020 36764363 10.1016/j.jad.2023.02.020

[CR25] Lyell DJ, Chambers AS, Steidtmann D et al (2012) Antenatal identification of major depressive disorder: a cohort study. Am J Obstet Gynecol 207(6):506.e1-506.e6. 10.1016/j.ajog.2012.09.030 23099192 10.1016/j.ajog.2012.09.030

[CR26] McKee K, Admon LK, Winkelman TNA et al (2020) Perinatal mood and anxiety disorders, serious mental illness, and delivery-related health outcomes, United States, 2006-2015. BMC Womens Health 20(1):150. 10.1186/s12905-020-00996-6 32703202 10.1186/s12905-020-00996-6 PMC7376899

[CR27] Michigan Department of Health and Human Services. Michigan PRAMS. 2017.

[CR28] Misra S, Jackson VW, Chong J et al (2021) Systematic review of cultural aspects of stigma and mental illness among racial and ethnic minority groups in the United States: implications for interventions. Am J Community Psychol 68(3–4):486–512. 10.1002/ajcp.12516 33811676 10.1002/ajcp.12516

[CR29] O’Mahen HA, Flynn HA (2008) Preferences and perceived barriers to treatment for depression during the perinatal period. J Womens Health 17(8):1301–1309. 10.1089/jwh.2007.0631 10.1089/jwh.2007.0631 18816202

[CR30] Parks AK, Hayman LL (2024) Unveiling the Strong Black Woman Schema—evolution and impact: a systematic review. Clin Nurs Res 33(5):395–404. 10.1177/10547738241234425 38439544 10.1177/10547738241234425

[CR31] Gaynes B, Gavin N, Meltzer-Brody S, et al. Perinatal Depression: Prevalence, Screening Accuracy, and Screening Outcomes. *Evidence report/technology assessment*. 2005;119(AHRQ Publication No. 05-E006–2.) 10.1037/e439372005-001 PMC4780910 15760246

[CR32] Platt LF, Fanning SC (2023) The strong Black woman concept: associated demographic characteristics and perceived stress among Black women. J Black Psychol 49(1):58–84. 10.1177/00957984221096211

[CR33] Pollack LM, Chen J, Cox S et al (2022) Healthcare utilization and costs associated with perinatal depression among Medicaid enrollees. Am J Prev Med 62(6):e333–e341. 10.1016/j.amepre.2021.12.008 35227542 10.1016/j.amepre.2021.12.008 PMC9247863

[CR34] PRAMS Phase 8 Questionnaire Topic Reference (2016).

[CR35] Rutter LA, Howard J, Lakhan P, Valdez D, Bollen J, Lorenzo-Luaces L (2023) I haven’t been diagnosed, but I should be"-insight into self-diagnoses of common mental health disorders: cross-sectional study. JMIR Form Res 7:e39206. 10.2196/39206 36637885 10.2196/39206 PMC9883736

[CR36] Sahebi A, Kheiry M, Abdi K, Qomi M, Golitaleb M (2024) Postpartum depression during the COVID-19 pandemic: An umbrella review and meta-analyses. Front Psychiatry 15:1393737. 10.3389/fpsyt.2024.1393737 39050914 10.3389/fpsyt.2024.1393737 PMC11266160

[CR37] Segovia LM, Neiman E, Gillespie SL, Jancsura MK, Anderson CM (2025) Prenatal and postpartum care experiences among Black birthing people in the United States: an integrative review. J Midwifery Womens Health 70(2):235–246 39539102 10.1111/jmwh.13705 PMC11980764

[CR38] Shulman HB, D’Angelo DV, Harrison L, Smith RA, Warner L (Oct2018) The Pregnancy Risk Assessment Monitoring System (PRAMS): overview of design and methodology. Am J Public Health 108(10):1305–1313. 10.2105/AJPH.2018.304563 30138070 10.2105/AJPH.2018.304563 PMC6137777

[CR39] Sidebottom A, Vacquier M, LaRusso E, Erickson D, Hardeman R (Feb2021) Perinatal depression screening practices in a large health system: identifying current state and assessing opportunities to provide more equitable care. Arch Womens Ment Health 24(1):133–144. 10.1007/s00737-020-01035-x 32372299 10.1007/s00737-020-01035-x PMC7929950

[CR40] Siu AL, USPSTF (2016) Screening for depression in adults: US preventive services task force recommendation statement. JAMA 315(4):380–387. 10.1001/jama.2015.18392 26813211 10.1001/jama.2015.18392

[CR41] Tabb KM, Dalton VK, Tilea A et al (2023) Trends in antenatal depression and suicidal ideation diagnoses among commercially insured childbearing individuals in the United States, 2008–2018. J Affect Disord 320:263–267. 10.1016/j.jad.2022.09.120 36179783 10.1016/j.jad.2022.09.120 PMC9675712

[CR42] Taouk LH, Matteson KA, Stark LM, Schulkin J (2018) Prenatal depression screening and antidepressant prescription: obstetrician-gynecologists’ practices, opinions, and interpretation of evidence. Arch Womens Ment Health 21(1):85–91. 10.1007/s00737-017-0760-7 28770341 10.1007/s00737-017-0760-7

[CR43] Trost SL, Beauregard JL, Smoots AN et al (2021) Preventing pregnancy-related mental health deaths: Insights from 14 US maternal mortality review committees, 2008–17. Health Aff Millwood 40(10):1551–1559. 10.1377/hlthaff.2021.00615 34606354 10.1377/hlthaff.2021.00615 PMC11135281

[CR44] Zivin K, Courant A (2024) Disparities in utilization and delivery outcomes for women with perinatal mood and anxiety disorders. J Psychiatr Brain Sci 9(2):e240003. 10.20900/jpbs.20240003 38817312 10.20900/jpbs.20240003 PMC11138136

[CR45] Zivin K, Pangori A, Zhang X et al (2024) Perinatal mood and anxiety disorders rose among privately insured people, 2008-20. Health Aff Millwood 43(4):496–503. 10.1377/hlthaff.2023.01437 38507649 10.1377/hlthaff.2023.01437 PMC11163973

[CR46] Zivin K, Zhang X, Tilea A, et al. US Perinatal Mood and Anxiety Disorder Trends among Privately Insured Individuals, 2008–2020. *Health Affairs*. 2024;In Press

[CR47] Luca D, Garlow N, Staatz C, Margiotta C, Zivin K. *Societal Costs of Untreated Perinatal Mood and Anxiety Disorders in the United States*. Issue Brief. 2019. https://www.mathematica-mpr.com/our-publications-and-findings/publications/societal-costs-of-untreated-perinatal-mood-and-anxiety-disorders-in-the-united-states 10.2105/AJPH.2020.305619 PMC7204436 32298167

[CR48] Centers for Disease Control and Prevention. *How Does CDC Identify Severe Maternal Morbidity?* 2023. https://www.cdc.gov/reproductivehealth/maternalinfanthealth/smm/severe-morbidity-ICD.htm

[CR49] Centers for Disease Control and Prevention. Pregnancy Risk Assessment Monitoring System (PRAMS): Data Methodology. 2024. Accessed December 12, 2024. https://www.cdc.gov/prams/php/methodology/index.html

[CR50] Hill L, Artiga S, Ranji U. Racial Disparities in Maternal and Infant Health: Current Status and Efforts to Address Them. *KFF Health News*. 2022. https://www.kff.org/racial-equity-and-health-policy/issue-brief/racial-disparities-in-maternal-and-infant-health-current-status-and-efforts-to-address-them/

[CR51] Avalos LA, Nance N, Iturralde E, et al. Racial-Ethnic Differences in Treatment Initiation for New Diagnoses of Perinatal Depression. *Psychiatric services (Washington, DC)*. 2022;10.1176/appi.ps.20220173 10.1176/appi.ps.20220173 PMC10084773 36226373

